# To Lyse or Not: The Role of Half‐Dose Thrombolysis in Right Ventricular Clot Associated With Sub‐Massive Pulmonary Embolism

**DOI:** 10.1002/ccr3.70404

**Published:** 2025-04-22

**Authors:** Nasreldin A. Hamza, Wasfy J. Hamad, Shamim K. Vakkulathil, Vimalraj Sundaram, Haidar M. Hadi, Abdulqadir J. Nashwan

**Affiliations:** ^1^ Critical Care Department Al‐Khor General Hospital, Hamad Medical Corporation Doha Qatar; ^2^ Cardiology Department Al‐Khor General Hospital, Hamad Medical Corporation Doha Qatar; ^3^ Nursing Department Hamad Medical Corporation Doha Qatar

**Keywords:** deep vein thrombosis, pulmonary embolism, right heart thrombi, right ventricular thrombus, thrombolytic therapy

## Abstract

Right heart thrombi are rare but carry a high mortality risk. Appropriate therapy remains unclear; options include anticoagulation, thrombolysis, and surgical thrombectomy. In this case, a 43‐year‐old male with DVT, RV thrombus, and bilateral pulmonary embolism responded well to half‐dose thrombolytic therapy, with complete thrombus resolution and no complications.

## Graphical abstract

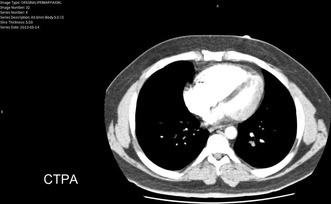

## 1 Introduction

RV thrombus is a rare finding usually found with concurrent emboli and associated with significant mortality [1]. Recognition of intracardiac thrombus and immediate intervention help prevent the disastrous consequences, which include sudden death [2]. The ideal management of intraventricular thrombi is not clear. Although there are a large number of clinical trials suggesting different treatment options, the existing evidence is controversial.

We present a case report addressing the uncertainty of using a half‐dose thrombolytic regimen. This approach has shown promising results in resolving thrombi, improving right heart function, and reducing the risk of bleeding.

## 2 Case History/Examination

A 43‐year‐old male with a history of uncontrolled diabetes mellitus, chronic smoking, and COPD/asthma (not previously investigated) presented with a 3‐day history of shortness of breath and cough, with no hemoptysis. There was no lower limb pain or swelling. Family history of deep vein thrombosis was noted. Upon admission, he was found to be hypoxic, with a blood oxygen saturation of 90% on room air. With the administration of 40% venturi support, saturation improved to 95%. Other vitals include blood pressure 150/98, pulse 118, temperature 37°C (Oral), and respiratory rate 23.

## 3 Methods (Differential Diagnosis, Investigations, and Treatment)

**FIGURE 1:**
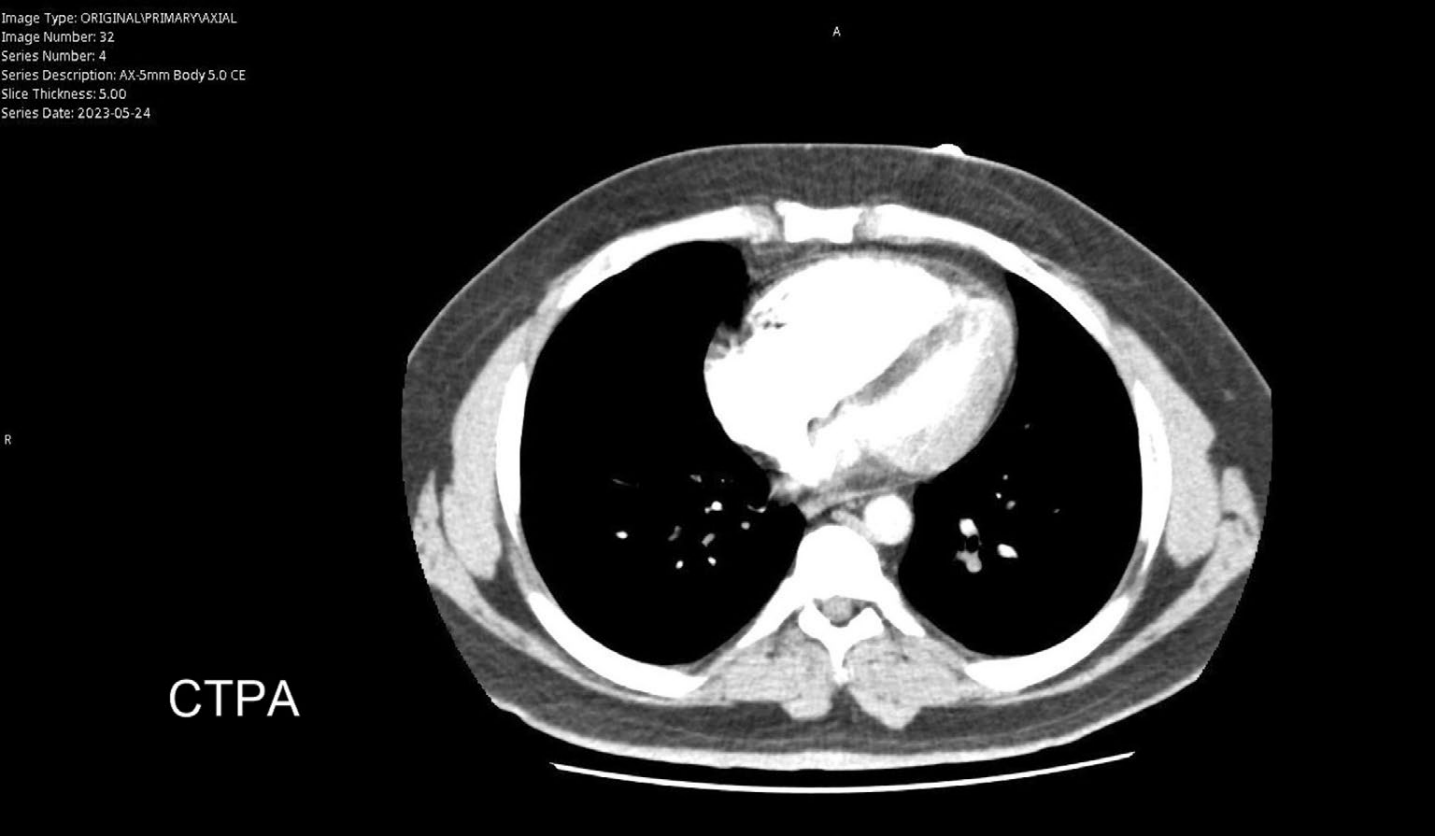
A chest CT angiogram revealed multiple filling defects in the distal main pulmonary artery and showed signs of right ventricular strain, evidenced by the dilatation of the main pulmonary artery (3.3 cm) and flattening of the interventricular septum toward the left ventricular cavity.

**FIGURE 2:**
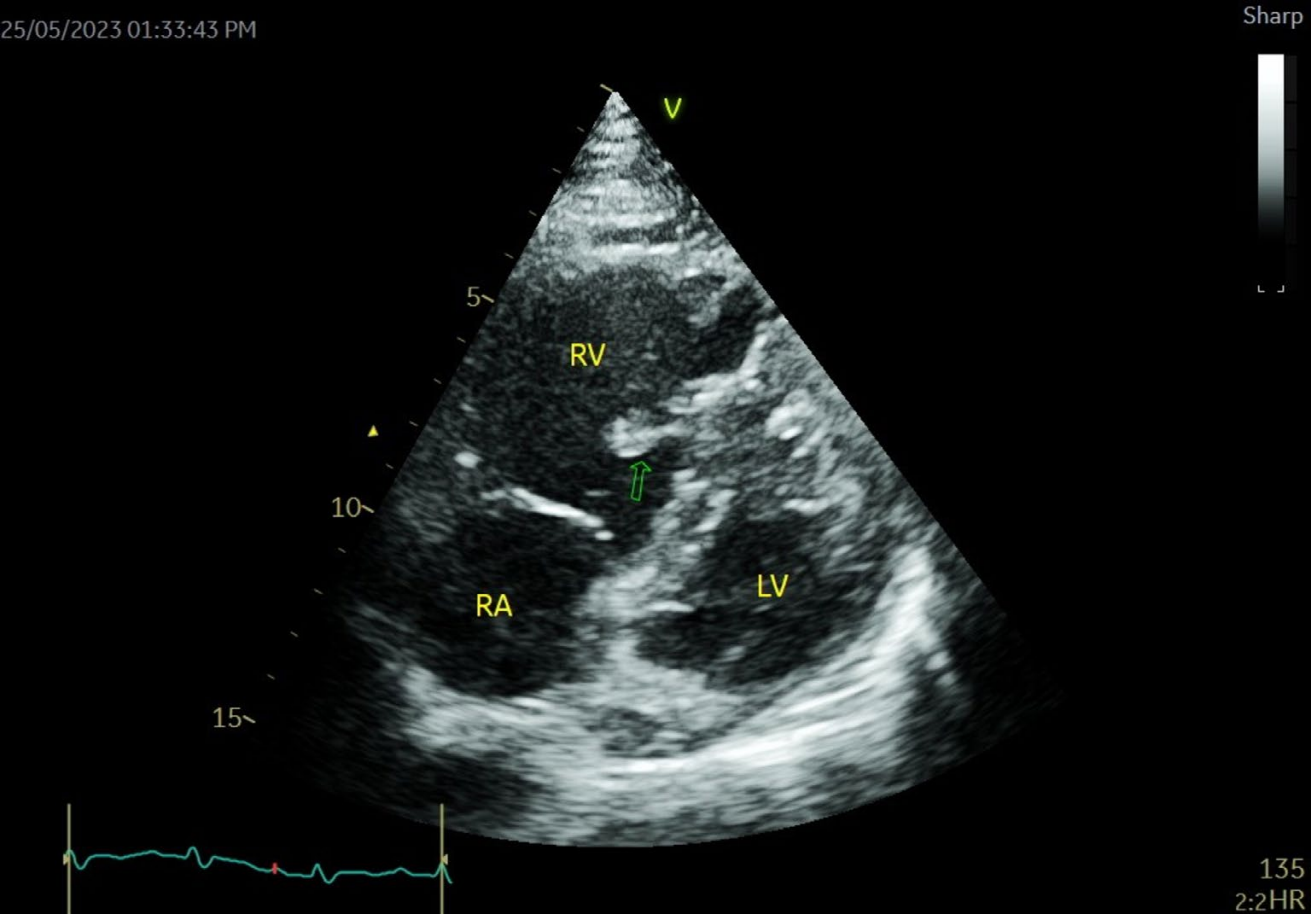
Dilated RV and evidence of a 1.5 × 2 cm mass (RV thrombus) attached to mid‐IVS from the RV side (arrow).

**FIGURE 3:**
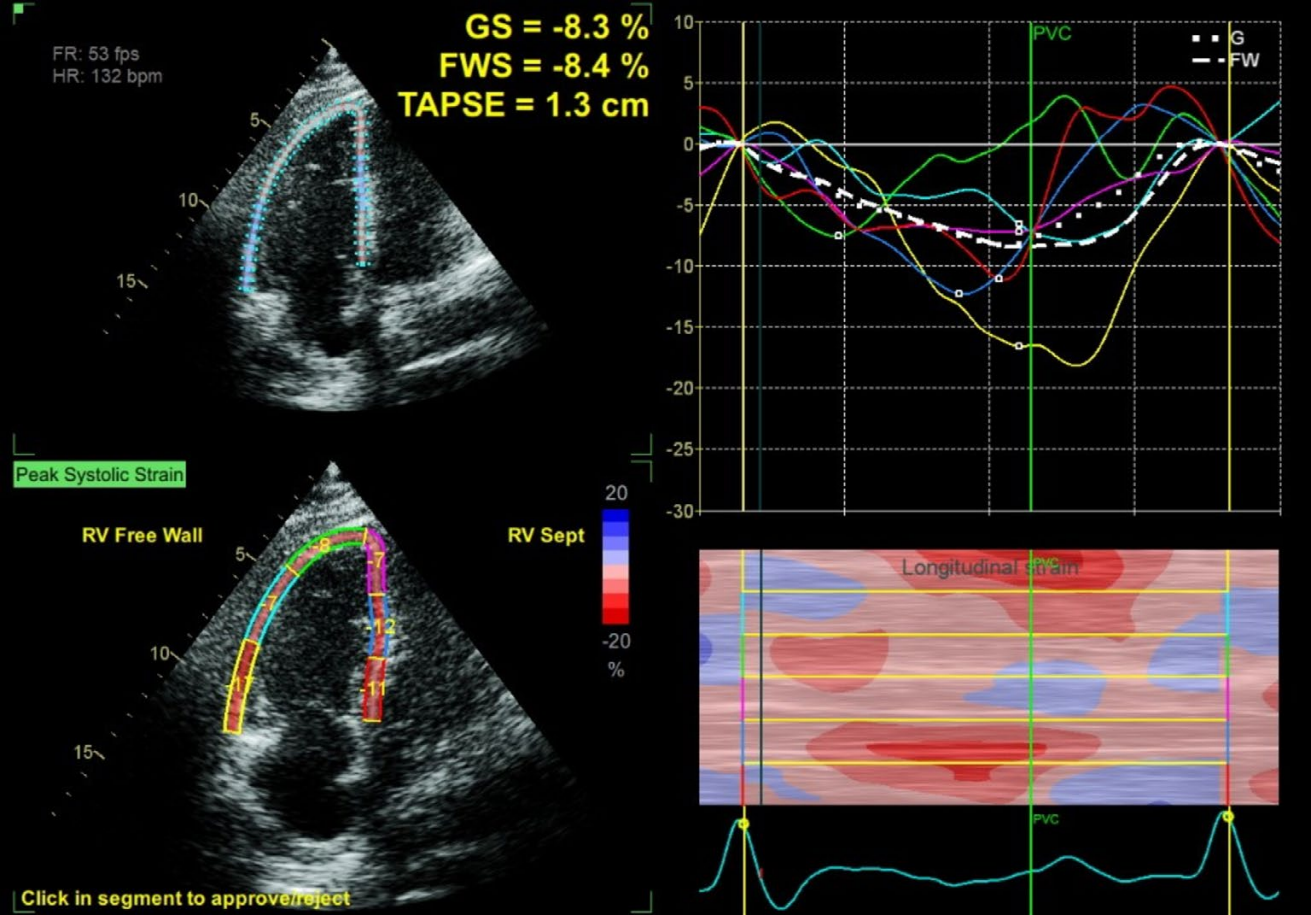
RV strain analysis showing evidence of RV dysfunction with RV global strain = −8.3%, FWS = −8.4%, and TAPSE = 1.3 cm.

Initial chest CT angiogram revealed multiple filling defects in the distal main pulmonary artery, descending and upper lobar arteries, extending into the lobar and segmental arteries, with signs of right ventricular strain, evidenced by the dilatation of the main pulmonary artery (3.3 cm) and flattening of the interventricular septum toward the left ventricular cavity (Figure 1). A Doppler ultrasound of the lower limbs showed a bilateral deep vein thrombus. Echocardiography conducted upon admission identified a moderately dilated right ventricle with moderately reduced function, along with a mobile thrombus attached to the mid‐right interventricular septal wall, severe pulmonary hypertension (RVSP 60 mmHg), and evidence of RV pressure overload (Figure 2). Further RV strain analysis confirmed impaired function, with global RV strain at −8.3%, RV free wall strain at −8.4%, and tricuspid annular plane systolic excursion at 1.3 cm (Figure 3).

**FIGURE 4:**
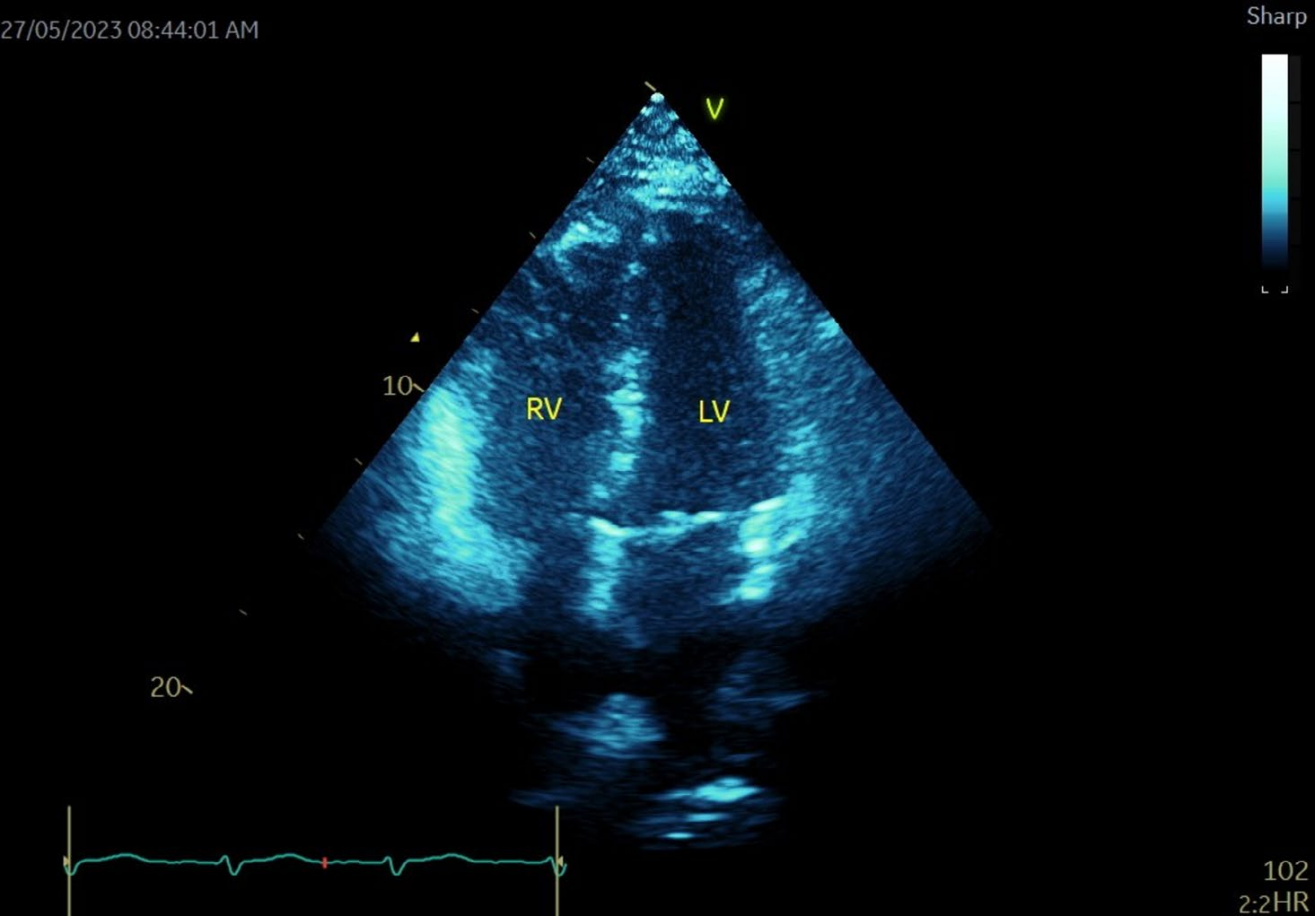
The complete resolution of RV thrombus post half‐dose lysis.

The patient was initially managed with oxygen therapy and intravenous heparin infusion to target an aPTT value of 50, adjusted from a baseline of 25. This was later switched to therapeutic enoxaparin at 120 mg twice daily. On the second day, due to the presence of the RV thrombus, lack of clinical improvement (persistent dyspnoea and requirement of oxygen 40% by a venturi mask, heart rate of 110, and respiratory rate of 20), and increasing trends in Pro BNP (1549 to 2048), troponin T (37 to 99), and D‐dimer (3.67 to 5.04), thrombolytic therapy with 50 mg alteplase administered intravenously over 2 h was initiated, followed by a return to heparin infusion (Figure 4). The cardiothoracic team was consulted, and they recommended continuing anticoagulation without intervention. The patient was subsequently transferred to a tertiary care center for further management.

## 4 Conclusion and Results (Outcome and Follow‐Up)

On the third day, a repeat echocardiogram showed the disappearance of the RV mobile thrombus, with mildly reduced RV function, moderate dilatation of the RV chamber, and an RVSP of approximately 45 mmHg. By the fourth day, Pro BNP had decreased to 274, and D‐dimer levels decreased to 4.13, though cardiac markers (troponin) remained elevated. The patient was discharged on the sixth day with a prescription for rivaroxaban 15 mg BID.

## 5 Discussion

Venous thromboembolism has a broad spectrum of clinical syndromes associated with varying clinical outcomes [1, 2]. Right heart thrombi (RHT) are uncommon but probably underdiagnosed in patients with pulmonary embolism [1]. Mobile right‐heart thrombi are detected in < 4% of unselected patients with PE [3, 4]; however, they can range from 2.6% to 18% [5].

RHT had a higher prevalence in patients with a history of chronic heart failure, cancer, immobilization, or recent major bleeding compared with those without RHT [2]. Three RHTs (A, B, and C) are described, suggesting specific etiologies [3]. Type A describes a highly mobile serpiginous thrombus, often trapped in right heart cavities, representing the result of a migration of thrombi [3]. Hence, type A thrombi are associated with DVT and PE. Type B thrombi are fixed, formed in situ, and associated with cardiac abnormalities [3]. Type C assumes intermediate characteristics [3]. Stasis in the dilated right heart due either to acute severe pulmonary embolism, pre‐existing congestive heart failure, or both seems to enhance the risk of RHT, regardless of whether it is due to in situ thrombosis or to entrapment of transiting thrombi [2].

There is a lack of comprehensive data on the prevalence, predictors, and prognostic significance of RHT in pulmonary embolism [2]. When compared to patients without RHT, patients with RHT are more hemodynamically compromised, with lower systolic blood pressure, higher heart rate, and more frequent hypoxemia and syncope [2]. A patent foramen ovale also increases the risk of ischemic stroke due to paradoxical embolism [4]. The literature review suggests that patients with acute pulmonary embolism and RHT had a significantly higher cumulative mortality than patients with acute pulmonary embolism without RHT [2]. Among low‐risk PE patients with RHT, the risk of death was about seven times higher than in patients without RHT [2]. Among patients with low and intermediate risk of pulmonary embolism (i.e., right ventricular dysfunction), those with RHT had an increased mortality compared with those without RHT [2]. Our patient was thus considered to have a higher mortality risk.

Primary treatment includes optimizing oxygenation and ventilation status, volume optimization, and vasopressors and inotropes as needed [4]. Further therapeutic options in these patients consist of anticoagulation, thrombolysis, or surgical embolectomy [2]. However, the optimal treatment of right heart thrombi remains unclear, as no randomized studies exist. Current available data show that in patients who received anticoagulation alone, the risk of death was about three times higher in those with RHT than in those without RHT [2]. Data also suggested that normotensive patients with PE and coexisting RHT had a threefold increased risk of short‐term death compared with patients without RHT [5]. It was thus concluded that anticoagulation is not enough as the sole treatment of pulmonary embolism with RHT [1], and the feasibility of thrombolysis was considered in our patient.

In normotensive patients with intermediate‐risk pulmonary embolism (PE), defined as the presence of RV dysfunction and elevated troponin levels, the impact of thrombolytic treatment was investigated in the Pulmonary Embolism Thrombolysis (PEITHO) trial, which showed a significant reduction in the risk of hemodynamic decompensation or collapse. Still, an increased risk of severe extracranial and intracranial bleeding paralleled this [4]. The MOPETT trial also demonstrated that low‐dose thrombolysis in patients with submassive pulmonary embolism significantly reduces the risk of pulmonary hypertension and improves outcomes with a lower risk of bleeding. The efficacy of “half‐dose” or “safe dose” thrombolytic therapy in the resolution of five cases of floating right heart thrombus (FRHT) was presented in a case series [6]. Here, half‐dose thrombolytic therapy (50 mg of alteplase) was administered to the patients and followed up with an echocardiogram that revealed complete resolution of the FRHT and considerable improvement in the right heart function with no bleeding events [6, 7]. Hence, in our case, we perform thrombolysis with half dose to further reduce the risk of bleeding.

Venous thromboembolism encompasses a range of clinical presentations and outcomes, with RHT being an underdiagnosed but significant complication. RHT can notably impact prognosis, leading to higher mortality and hemodynamic instability. Optimal treatment for RHT remains debated due to the lack of randomized studies, though anticoagulation, thrombolysis, and surgical options are considered. Thrombolysis, including “half‐dose” regimens, has shown promising results in resolving RHT while minimizing bleeding risks, as demonstrated in our case. This underscores the necessity for tailored therapeutic approaches based on individual risk assessments and the need for further research to establish standardized treatment protocols for RHT in PE.

## Author Contributions

**Nasreldin A. Hamza:** writing – original draft, writing – review and editing. **Wasfy J. Hamad:** writing – original draft, writing – review and editing. **Shamim K. Vakkulathil:** writing – original draft, writing – review and editing. **Vimalraj Sundaram:** writing – original draft, writing – review and editing. **Haidar M. Hadi:** writing – original draft, writing – review and editing. **Abdulqadir J. Nashwan:** writing – original draft, writing – review and editing.

## Ethics Statement

Following local or national guidelines, ethical approval was not required for this case. However, the Medical Research Center at Hamad Medical Corporation (MRC‐04‐24‐475) approved the case for publication.

## Consent

Written informed consent was obtained from the patient to publish this report in accordance with the journal's patient consent policy.

## Conflicts of Interest

The authors declare no conflicts of interest.

## Data Availability

All data generated or analyzed in this study are included in this published article. Further inquiries can be directed to the corresponding author.
